# Systematic review and bibliometric analysis of African anesthesia and critical care medicine research part II: a scientometric analysis of the 116 most cited articles

**DOI:** 10.1186/s12871-021-01246-4

**Published:** 2021-01-21

**Authors:** Ulrick Sidney Kanmounye, Joel Noutakdie Tochie, Aimé Mbonda, Cynthia Kévine Wafo, Leonid Daya, Thompson Hope Atem, Arsène Daniel Nyalundja, Daniel Cheryl Eyaman

**Affiliations:** 1 Department of Research, Association of Future African Neurosurgeons, Yaounde, Cameroon; 2 Department of Neurosurgery, Faculty of Medicine, Bel Campus University of Technology, Kinshasa, Democratic Republic of Congo; 3 Department of Anesthesiology and Critical Care Medicine, Faculty of Medicine and Biomedical Sciences, University of Yaounde I, Yaounde, Cameroon; 4 Human Research Education and Networking, Yaounde, Cameroon; 5 Faculty of Medicine and Biomedical Sciences, University of Yaounde I, Yaounde, Cameroon; 6 Surgery Unit, District Hospital of Batouri, Batouri, Cameroon; 7 Department of Research, International Student Surgical Network, Yaounde, Cameroon; 8 Department of Internal Medicine, Faculty of Medicine, Bel Campus University of Technology, Kinshasa, Democratic Republic of Congo; 9 Faculty of Medicine, Catholic University of Bukavu, Bukavu, Democratic Republic of Congo

**Keywords:** Africa, Anesthesia, Bibliometrics, Global anesthesia, Research

## Abstract

**Background:**

Scientometrics is used to assess the impact of research in several health fields, including Anesthesia and Critical Care Medicine. The purpose of this study was to identify contributors to highly-cited African Anesthesia and Critical Care Medicine research.

**Methods:**

The authors searched Web of Science from inception to May 4, 2020, for articles on and about Anesthesia and Critical Care Medicine in Africa with ≥2 citations. Quantitative (H-index) and qualitative (descriptive analysis of yearly publications and interpretation of document, co-authorship, author country, and keyword) bibliometric analyses were done.

**Results:**

The search strategy returned 116 articles with a median of 5 (IQR: 3–12) citations on Web of Science. Articles were published in Anesthesia and Analgesia (18, 15.5%), World Journal of Surgery (13, 11.2%), and South African Medical Journal (8, 6.9%). Most (74, 63.8%) articles were published on or after 2013. Seven authors had more than 1 article in the top 116 articles: Epiu I (3, 2.6%), Elobu AE (2, 1.7%), Fenton PM (2, 1.7%), Kibwana S (2, 1.7%), Rukewe A (2, 1.7%), Sama HD (2, 1.7%), and Zoumenou E (2, 1.7%). The bibliometric coupling analysis of documents highlighted 10 clusters, with the most significant nodes being Biccard BM, 2018; Baker T, 2013; Llewellyn RL, 2009; Nigussie S, 2014; and Aziato L, 2015. Dubowitz G (5) and Ozgediz D (4) had the highest H-indices among the authors referenced by the most-cited African Anesthesia and Critical Care Medicine articles. The U.S.A., England, and Uganda had the strongest collaboration links among the articles, and most articles focused on perioperative care.

**Conclusion:**

This study highlighted trends in top-cited African articles and African and non-African academic institutions’ contributions to these articles.

**Supplementary Information:**

The online version contains supplementary material available at 10.1186/s12871-021-01246-4.

## Background

Scientometrics is the branch of bibliometrics that analyzes the impact of peer-reviewed articles and scientific journals [1]. The impact of peer-reviewed articles can be evaluated both quantitatively and qualitatively. Of the two methods, quantitative scientometrics is more common. Some of the most common quantitative measures include the *h*-index, i10-index, g-index, or Page-Rank index. These indices are commonly used to measure the academic output and rank researchers and academic institutions. Unlike quantitative measures, qualitative scientometrics is less commonly used [2]. Qualitative scientometrics identifies articles, researchers, academic institutions, and themes of a field, and it maps the interactions between these individual items.

Anesthesia and critical care medicine (A.C.C.M.), like other health-related fields, use scientometrics to evaluate scholarly impact [3–6]. However, little is known about the impact of A.C.C.M. research. We aimed to identify the most impactful studies, the greatest contributors, and emerging themes in A.C.C.M. with quantitative and qualitative methods.

## Methods

### Defining A.C.C.M. research

In this systematic review of A.C.C.M. research with scientometric analysis, A.C.C.M. research was defined as research on the practice of A.C.C.M. in Africa irrespective of the authors’ academic affiliation or nationality. The study protocol was developed and posted online (htttp://doi:10.13140/RG.2.2.28999.32167).

### Search strategy and data sources

A systematic search of articles reporting the practice of A.C.C.M. in Africa was performed. The relevant articles were identified using a broad search strategy to capture terms associated with “Anesthesia,” “Critical care medicine,” and “Africa.” The search was done on Web of Science Core Collection, Arabic Citation Index, Russian Science Citation Index, Chinese Science Citation Database, Data Citation Index, BIOSIS Citation Index, and SciELO Citation Index. The advanced search strategy (Additional File 1) was developed by the first author, who has received formal training in information management (U.S.K.). All articles published from inception to May 4, 2020, were included irrespective of the language. All articles with ≥2 citations were included.

### Screening and data extraction

Each title and abstract was screened in Rayyan (Qatar Computing Research Institute, Qatar) by two authors (U.S.K. and J.N.T.), and the two authors resolved conflicts. The eligible articles were exported as text files then uploaded unto Bibexcel (Bibexcel, Austria) for bibliometric citation analysis. Next, the data from Bibexcel were uploaded on VOSviewer (University of Leiden, Netherlands) for content analysis. Publication trends were visualized as a bar chart, while bibliometric coupling, co-authorship, author country, and keyword were visualized as social network maps. The H-index values were calculated from the 116 A.C.C.M. articles. Also, first author affiliation data were extracted, and the articles were grouped into three categories: 1) Articles by first authors from African institutions (without non-African affiliations), 2) Articles by first authors with dual affiliations at African and non-African institutions, and 3) Articles by first authors from non-African institutions. Ethical clearance was not necessary for this study.

## Results

We found 116 articles on or about African A.C.C.M. with ≥2 citations. The 116 articles had 5 (IQR: 3-12) median Web of Science citations and 5 (IQR: 3–13) median citations when considering additional citation sources (BIOSIS, Chinese Science Citation, Data Citation, Russian Science, and SCIELO). The median usage count (since 2013) was 2 (IQR: 1–4).

The articles were published in Anesthesia and Analgesia (18, 15.5%), World Journal of Surgery (13, 11.2%), South African Medical Journal (8, 6.9%), and Canadian Journal of Anesthesia (6, 5.2%) (Table 1).

**Table 1:** List of journals with more than two articles among the most cited African anesthesia articles

Journal	Number of articles (***N*** = 116, %)
1. Anesthesia and Analgesia	18 (15.5)
2. World Journal of Surgery	13 (11.2)
3. South African Medical Journal	8 (6.9)
4. Canadian Journal of Anesthesia	6 (5.2)
5. Pediatric Anesthesia	5 (4.3)
6. Anaesthesia	4 (3.4)
7. Journal of Clinical Anesthesia	4 (3.4)
8. Nigerian Journal of Clinical Practice	4 (3.4)
9. B.M.C. Anesthesiology	3 (2.6)
10. Surgery	3 (2.6)
11. Anesthesiology	2 (1.7)
12. Annales Françaises d’Anesthésie et de Réanimation	2 (1.7)
13. British Journal of Anaesthesia	2 (1.7)
14. Journal of Tropical Medicine and Hygiene	2 (1.7)
15. Pan African Medical Journal	2 (1.7)
16. PLoS One	2 (1.7)
17. Southern African Journal of Anaesthesia and Analgesia	2 (1.7)

**Fig. 1:**
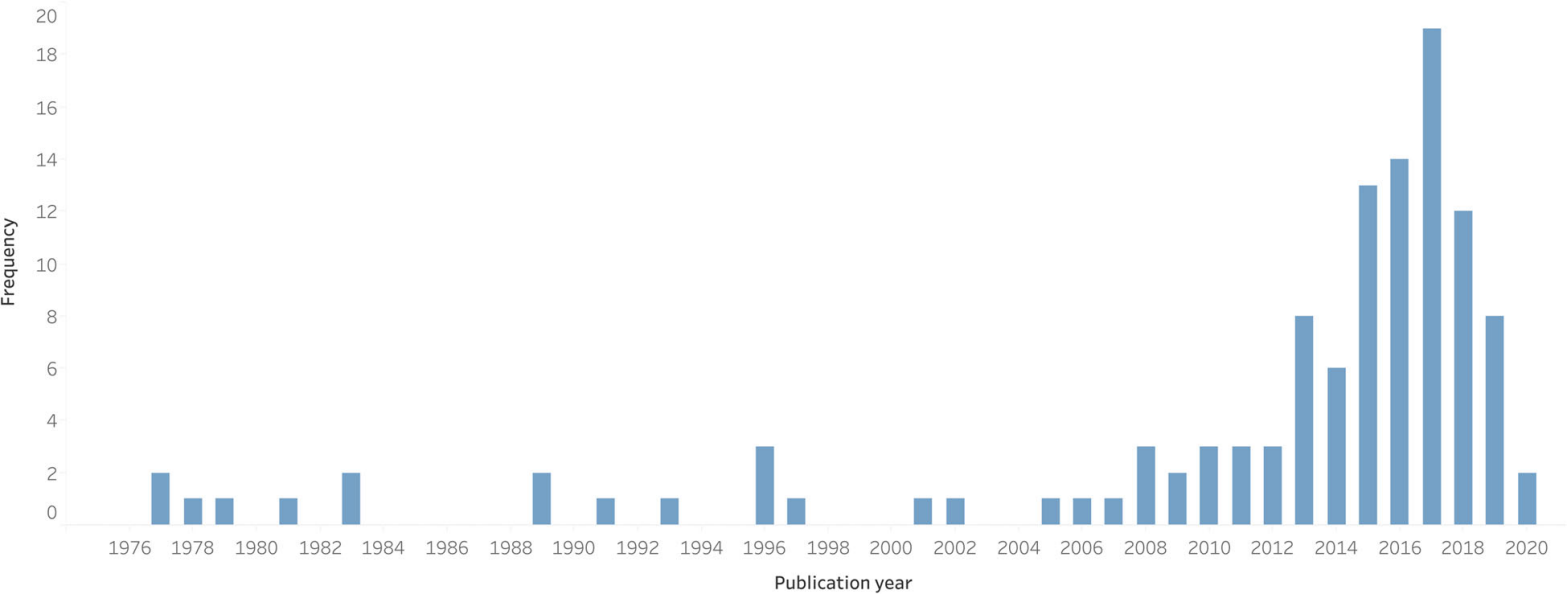
Time-trend of the most cited African anesthesia publications

More than half (74, 63.8%) of the articles were published in 2013 or later (Fig. 1). Seven authors had more than 1 article in the top 116 articles: Epiu I (3, 2.6%, U.S.A.), Elobu AE (2, 1.7%, Uganda), Fenton PM (2, 1.7%, Malawi), Kibwana S (2, 1.7%, Ethiopia), Rukewe A (2, 1.7%, Nigeria), Sama HD (2, 1.7%, Togo), and Zoumenou E (2, 1.7%, Benin).

**Fig. 2:**
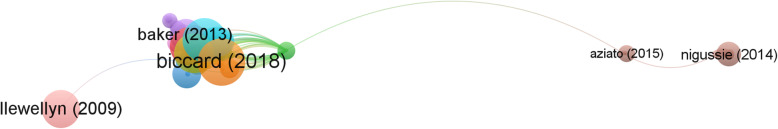
Bibliographic coupling - document analysis of the top 116 most cited articles on African anesthesia and critical care medicine

The bibliometric coupling analysis of documents identified 10 clusters with a total link strength of 1293 from 84 items and 766 links. The largest nodes were Biccard BM, 2018 (South Africa) [7]; Baker T, 2013 (Sweden) [8]; Llewellyn RL, 2009 (South Africa) [9]; Nigussie S, 2014 (Ethiopia) [10]; and Aziato L, 2015 (Ghana) [11] (Fig. 2).

**Fig. 3:**
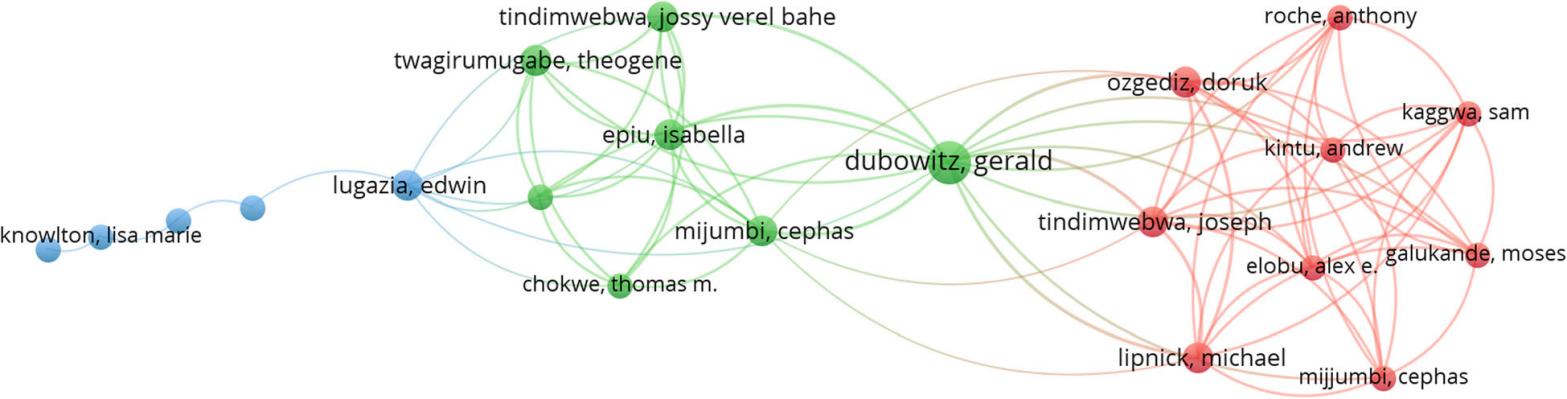
Co-author analysis of the top 116 most cited articles on African anesthesia and critical care medicine

Dubowitz G (5, U.S.A.) and Ozgediz D (4, U.S.A.) had the highest H-index among the authors referenced by the most-cited African anesthesia articles. Seventy-two authors had an H-index of 2 or higher (Table 2). Co-author analysis of the highest contributors’ articles revealed 21 co-authors grouped into 3 clusters and connected by 80 links (total link strength 156). Dubowitz G (46 total link strength, U.S.A.), Lugazia E (33 total link strength, Tanzania), Zoumenou E (29 total link strength, Benin), Lipnick M (27 total link strength, U.S.A.), Meara J (27 total link strength, U.S.A.), Ozgediz Z (27 total link strength, U.S.A.), and Tindimwebwa J (27 total link strength, Uganda) had the collaborations with the strongest links (Fig. 3).

**Table 2:** Backward citation analysis of the most cited African anesthesia studies. Only authors with an H-index greater than or equal to 2 are shown. Backward citation analysis shows the authors regualrly referenced by the 116 most cited A.C.C.M. articles. The H-index is calculated from the 116 A.C.C.M. articles only and does not represent the authors lifetime H-index

Author	H-Index	Citation sum within H-core	All citations	All articles
Dubowitz G	5	115	119	6
Ozgediz D	4	82	82	4
Maman AFOB	3	63	65	4
Twagirumugabe T	3	43	43	3
Ttendo SS	3	15	15	3
White MC	3	30	30	3
Lugazia E	3	82	82	3
Pollach G	3	21	23	4
Shrime MG	3	43	43	3
Mijumbi C	3	79	79	3
Rukewe A	3	15	15	3
Chobli M	3	61	63	4
Tindimwebwa J.V.B.	3	44	44	3
Kaggwa S	3	75	75	3
Namboya F	3	21	23	4
Tindimwebwa J	3	75	75	3
Epiu I	3	44	44	3
Zoumenou E	3	61	64	4
Lipnick M	3	75	75	3
Firth PG	3	15	15	3
Measures E	2	15	15	2
Baxter LS	2	24	26	3
Meara J	2	47	47	2
Roche A	2	35	35	2
Stekelenburg J	2	4	4	2
Close KL	2	24	24	2
Nelson BD	2	21	21	2
McQueen KAK	2	81	81	2
Elobu AE	2	35	35	2
Thwaites V	2	5	5	2
Ravelojaona VA	2	24	24	2
Evans F	2	27	27	2
McEvoy MD	2	14	16	3
McQueen K	2	87	87	2
Thomas J	2	6	6	2
Newton MW	2	14	16	3
Ndarugirire F	2	39	39	2
Bruno E	2	24	24	2
Bulamba F	2	5	5	2
Mkandawire N	2	9	9	2
Scribante J	2	8	8	2
Sama HD	2	92	94	3
Sandberg WS	2	14	14	2
Burke TF	2	21	21	2
Herbert A	2	24	24	2
Mijjumbi C	2	35	35	2
Sileshi B	2	14	16	3
Enright A	2	39	39	2
Ismailova F	2	80	82	3
Bould MD	2	18	20	3
Missair A	2	12	12	2
Shotwell MS	2	14	14	2
Chokwe TM	2	39	39	2
Towey RM	2	15	15	2
Fenton PM	2	30	30	2
Galukande M	2	35	35	2
van Roosmalen J	2	4	4	2
Edgcombe H	2	5	5	2
Preston MA	2	12	12	2
Was A	2	12	12	2
Andriamanjato HH	2	24	24	2
Kintu A	2	35	35	2
Kinnear JA	2	18	20	3
Perrie H	2	8	8	2
Kibwana S	2	4	4	2
Downing JW	2	12	12	2
Ngumi Z.W.W.	2	89	89	2
Rakotoarison HN	2	24	24	2
Fatiregun A	2	7	7	2
Livingston P	2	31	31	2
Knowlton LM	2	121	121	2
Lokossou T	2	29	29	2

**Fig. 4:**
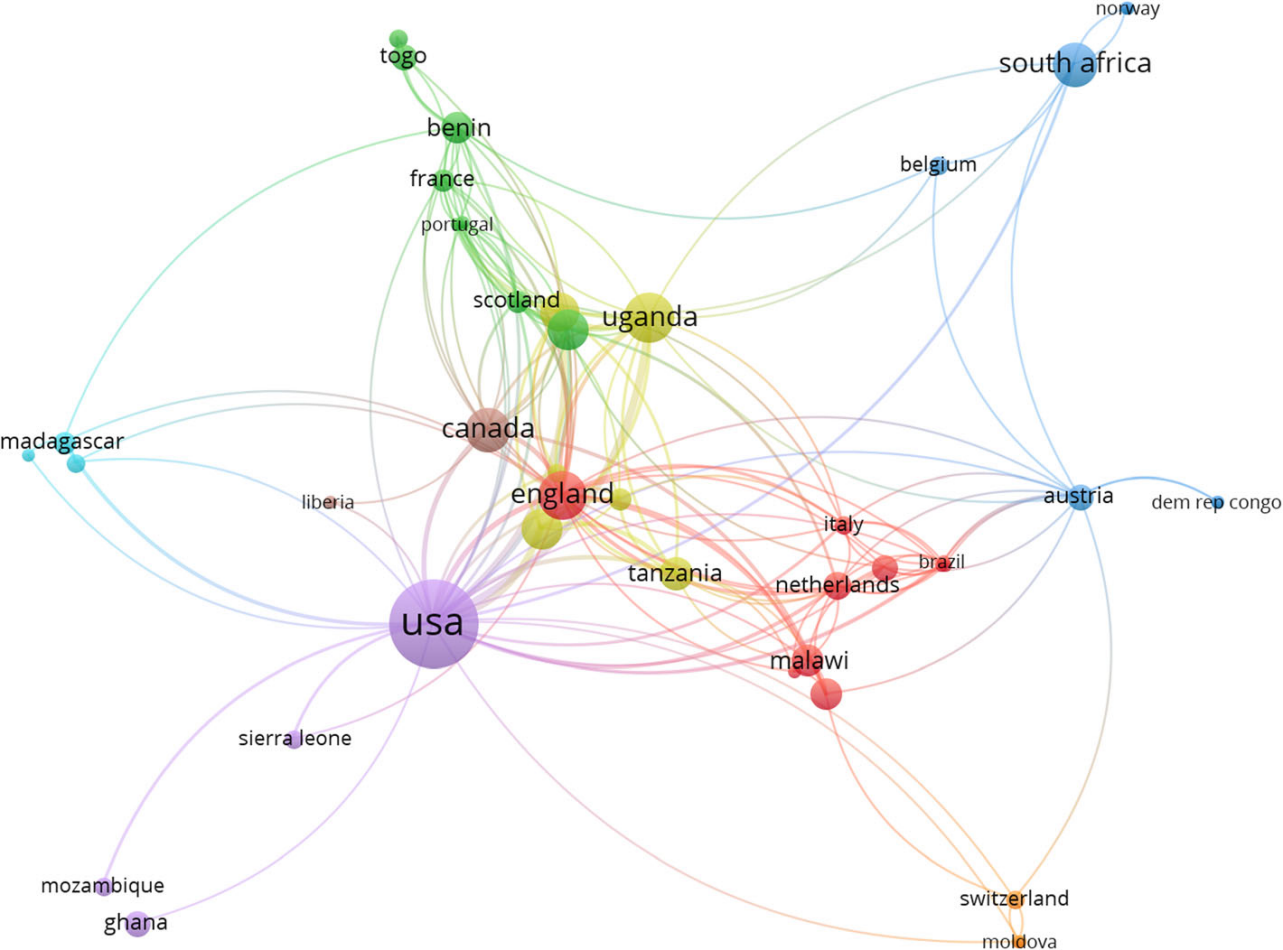
Author affiliation analysis of the top 116 most cited articles on African anesthesia and critical care medicine

First authors with affiliations at African institutions contributed 57 (49.1%) articles while 52 (44.8%) articles were by first authors affiliated with non-African institutions, and seven (6.0%) articles were from first authors with affiliations at African and non-African institutions. First and co-authors were affiliated with institutions in 43 countries. The countries were organized into 8 clusters with 176 collaborations (links) and a total link strength of 271. Sixteen of these countries contributed substantially to the most cited A.C.C.M. research: 12 (75.0%) African, 2 (12.5%) North American, and 2 (12.5%) European. The U.S.A. had the highest number of collaborations (86 total link strength, 46 documents, 645 citations), followed by England (71 total link strength, 15 documents, 194 citations), Uganda (65 total link strength, 16 documents, 278 citations), Canada (54 total link strength, 13 documents, 304 citations), and Kenya (47 total link strength, 11 documents, 198 citations). Authors affiliated with South African institutions contributed to 13 articles and accumulated 210 citations (32 total link strength) (Fig. 4).

**Fig. 5:**
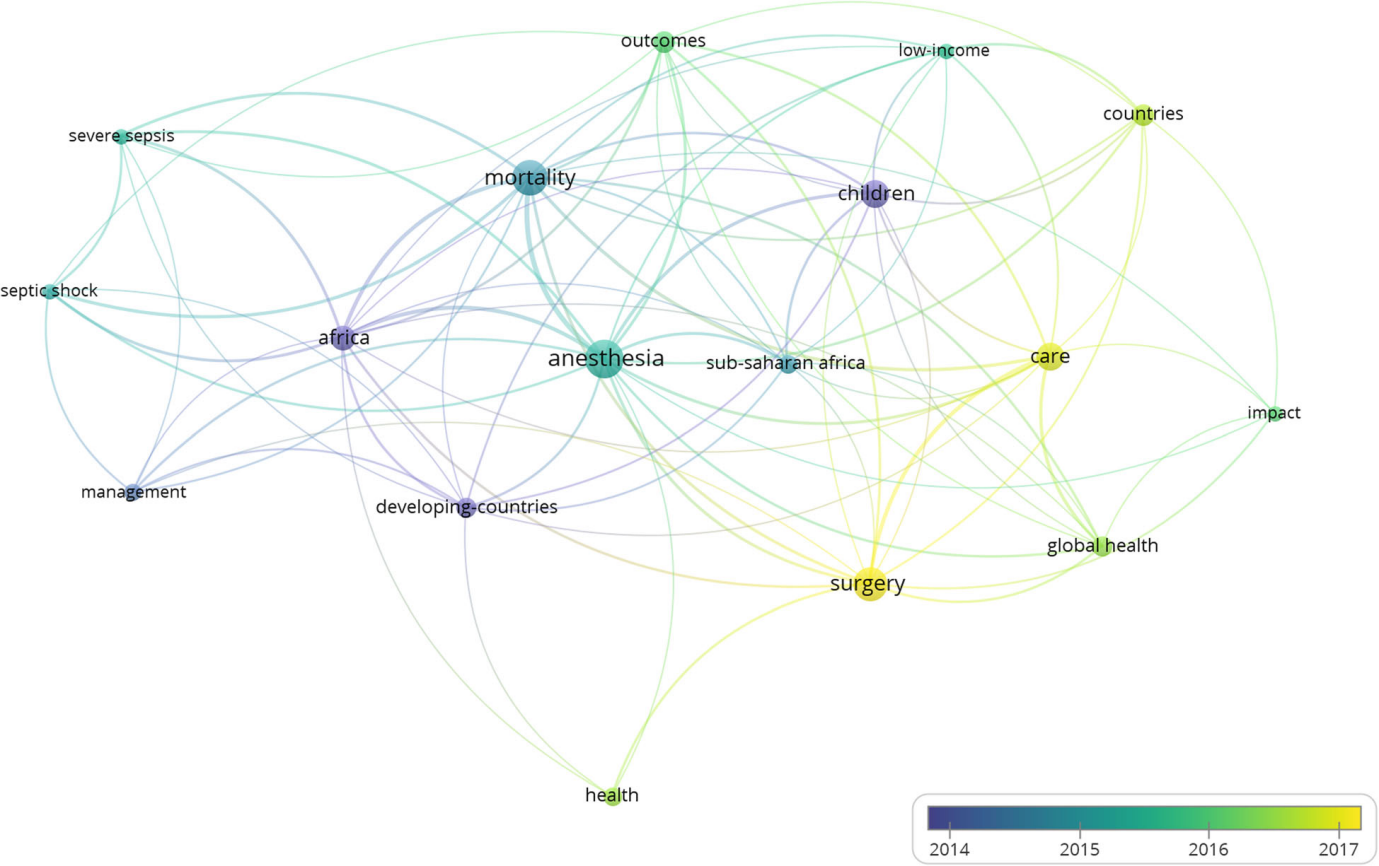
Co-occurrence analysis of the top 116 most cited articles on African anesthesia and critical care medicine

The keywords were organized into 4 clusters of 17 items with 85 links and a total link strength of 208. The four clusters covered perioperative care in low-resource settings, especially regarding children and sepsis (Fig. 5).

## Discussion

This systematic review with scientometric analysis is the first study to identify trends, themes, and contributors of the most cited A.C.C.M. research. Anesthesia and Analgesia (15.5%) had the most articles among the 116 studies. The first African journal, South African Medical Journal (6.9%), ranked third. The Southern African Journal of Anaesthesia and Analgesia (1.7%) was the first specialty African journal in eleventh place ex aequo. Epiu I had the greatest number of first author articles (2.6%), and the African Surgical Outcomes Study by Biccard et al. [7] was among the most influential papers. Also, Dubowitz had the highest H-index among the top 116 cited articles and his article on the Global anesthesia workforce cirsis [12] was regularly referenced by the most cited A.C.C.M. articles.

### Citations

Top-cited A.C.C.M. articles had lower scientometric measures than similar research from other regions [5]. Citation metrics are influenced by the time since publication and visibility. Most top-cited A.C.C.M. articles were published in 2013 or later, and a significant proportion of articles were published in prominent journals. For example, the most cited article, published by the African Perioperative Research Group in The Lancet, totaled 87 citations in two years [7]. The impressive citation metrics can be attributed to its publication in a high-impact factor journal but equally to its relevance and novelty. Biccard et al. [7] led the largest multicentre prospective study of 7-day postoperative mortality in Africa (25 African countries, 247 hospitals, and 11,422 patients), and they identified perioperative disparities between surgical specialties and African regions [7]. This African research collaborative set a precedent for high-impact clinical research in Africa, and we anticipate there will be similar initiatives and impact in the next few years.

Collaborations between African authors and institutions were less common than non-African and African collaborations. A.C.C.M. research collaborations will benefit greatly from partnering with African authors and institutions that have experience publishing high-impact research. These include Lugazia E (Tanzania), Zoumenou E (Benin), Tindimwebwa J (Uganda), Elobu AE (Uganda), Kibwana S (Ethiopia), Rukewe A (Nigeria), Sama HD (Togo), and Biccard B (South Africa).

While most first authors of top-cited A.C.C.M. articles were affiliated with African institutions, non-African academic institutions had more top-cited articles and greater citation metrics than African academic institutions. This observation is consistent with other reports. Global North researchers and institutions have higher citation metrics than Global South researchers and institutions [13, 14]. Moreover, African researchers have smaller scholarly outputs within the Global South than their counterparts from the other regions [15, 16]. The growth of scholarly productivity in Africa is stunted by numerous factors. Lack of funding and administrative support is the most commonly cited barriers to scholarly productivity in Africa [17]. These barriers forestall the design and publication of high quality (large sample, multicentric, prospective, randomized, and blinded) clinical research on the continent [4, 5]. Another consequence of the lack of funding is decreased visibility. Open access increases article visibility and citation; however, open access publication costs in some high-impact factor journals can be prohibitive [16–18]. Fortunately, an increasing number of high-impact factor journals offer open access fee waivers to authors from low-income countries and reductions for authors from lower- and upper-middle-income countries [19]. African authors from these middle-income countries who cannot afford reduced open-access costs either opt for a subscription-based journal or a less expensive journal [18].

The majority of top-cited A.C.C.M. articles were published in non-African journals. Local journals have lower or no publication fees but tend to have smaller readerships and impact factors [19]. As a result, African authors often have to choose between decreased visibility and expensive fees. Authors can increase their articles’ visibility by designing and disseminating visual abstracts, organizing journal clubs, and writing op-eds on their manuscripts [18, 20, 21]. Local journals should encourage A.C.C.M. authors to organize the post-publication activities mentioned above by providing toolkits and post-publication services.

### Keywords

The theme of the most cited A.C.C.M. research transitioned from critical care to anesthesia. This transition is demonstrated in Fig. 5 by the blue (older) sepsis-related keywords to the yellow (newer) anesthesia keywords. Sepsis is the most common cause of death from infectious diseases, and Africa has an enormous burden of infectious diseases [22–24]. In particular, the African region is among the most affected by the human immunodeficiency virus and *Mycobacterium tuberculosis*, the first and second causes of sepsis in Africa, respectively [23, 25]. Moreover, sepsis is responsible for USD 10–469 billion in financial loss among African families and states [26]. Despite the considerable clinical and financial burden of sepsis in Africa, it remains under-reported and under-researched [22]. The nodes of sepsis were smaller and less connected than those of anesthesia. Given the burden of sepsis and a limited number of top-cited sepsis-related articles, we suggest that A.C.C.M. stakeholders promote more novel and collaborative sepsis research.

This scientometric analysis equally highlights the need to increase the visibility of articles on other aspects of critical medicine. For example, research on the other components of the care continuum such as surveillance, prevention, prehospital care, and rehabilitation.

Although the terms “global health,” “low-income countries,” and “developing countries” were prominent, there was no noticeable “global anesthesia” node. It appears that global anesthesia research is accessible in Africa, but the term “global anesthesia” is not commonly used. Global anesthesia is a growing field that studies and advocates universal access to safe, timely, and affordable anesthesia care [27, 28]. 2010 was a marquee year for global anesthesia as Dubowitz et al. highlighted specialist workforce shortage in low-and middle-income countries and its impact on patient outcomes [12]. In the same year, McQueen published two articles on global anesthesia [29, 30]. While the three global anesthesia articles were not focused on African anesthesia, they inspired research in the region. Dubowitz et al. ‘s high H-index supports this claim (Table 2). A decade after the seminal global anesthesia studies publication, the term does not appear among the most influential keywords in African anesthesia research. Further research is needed to understand this phenomenon.

Biccard et al. [31] have proposed an agenda for A.C.C.M. research composed of ten priorities. Four of the ten priorities are health systems research, and two are clinical. The priorities include: A.C.C.M. education, service delivery, peripartum hemorrhage, non-technical skills, infrastructure, context-specific evidence-based practice, economic analyses, information management, quality improvement, and perioperative outcomes [31]. Other than perioperative outcomes (represented by the keywords “mortality” and “surgery”), the remaining research priorities set by Biccard et al. [31] do not appear among the most cited African A.C.C.M. studies.

### Contributions of non-African research institutions

The U.S.A. and the U.K. contributed significantly to the most-cited A.C.C.M. research. In addition, institutions from these countries were central to collaborations between African and non-African academic institutions. On the one hand, this finding highlights the benefit and magnitude of collaboration between Global North and African institutions. On the other hand, it draws attention to the lower representation of African researchers and institutions among the most cited A.C.C.M. studies. 55.1% of first authors were affiliated with an African institution. This proportion is greater than that reported in a recent systematic review of global health research in Africa. Hedt-Gauthier et al. found that 68.3% of papers had a collaborator from the Global North, and only 23.0% of first authors were local researchers [32]. The A.C.C.M. results are therefore encouraging; however, as we promote greater representation of local authors in A.C.C.M. research, we must avoid practices such as gift authorship that will undermine African researchers’ contributions. African researchers should be involved early on in A.C.C.M. collaborative research so they can contribute significantly and deserve first and last authorship positions.

### Limitations

There are several limitations to the present study. First, the definition of African A.C.C.M. research excludes articles by African researchers on the practice of Anesthesia in non-African countries. Including such studies would have been difficult and incomplete because of the inability to identify African researchers if they have a non-African affiliation. The publication focuses on A.C.C.M. practice in a non-African region. Next, few African journals can be found on the major search and citation databases. As a result, our decision to search major citation databases might have missed a significant proportion of articles on local A.C.C.M. practice. However, we wish to note that articles that are not found in one of the major citation databases are less likely to have citation data.

## Conclusion

This study is the first comprehensive scientometric analysis of African A.C.C.M. research. Top-cited A.C.C.M. research was published in reputable journals, and most articles had authors affiliated with African institutions. Most articles focused on perioperative care, and sepsis in Africa and non-African countries contributed significantly to citations and collaborations. Future research should compare A.C.C.M. research practices to identify the mechanisms adopted by high performers to replicate them in lower-performing regions.

## Supplementary Information

**Additional file 1.** Search Strategy.

## Data Availability

The datasets used and/or analyzed during the current study are available from the corresponding author on reasonable request.

## Abbreviation

A.C.C.M: Anesthesia and critical care medicine
