# Supplementary material for: Systematic review and bibliometric analysis of African anesthesia and critical care medicine research part II: a scientometric analysis of the 116 most cited articles
Source: BMC Anesthesiol. 2021 Jan 21;21:24. doi: 10.1186/s12871-021-01246-4 (PMC7819185; doi:10.1186/s12871-021-01246-4)
Supplement: Supplementary file 1 — Additional file 1. Search Strategy. [file 12871_2021_1246_MOESM1_ESM.docx]

**Additional File 1**

**Web of Science Search strategy**

TS=(anesthe*  AND africa)  OR TI=((Anesthe*  OR periopera*  OR postopera* or preopera*)  AND ("Africa"  OR "subsaharan Africa"  OR "Sub-Saharan Africa"  OR Algeria  OR Angola  OR Benin  OR Botswana  OR Burkina Faso  OR Burundi  OR Cabo Verde  OR Cameroon  OR Central African Republic  OR Chad  OR Comoros  OR Congo  OR Cote d'Ivoire  OR Democratic Republic of the Congo  OR Djibouti  OR Egypt  OR Equatorial Guinea  OR Eritrea  OR Eswatini  OR Ethiopia  OR Gabon  OR Gambia  OR Ghana  OR Guinea  OR Guinea-Bissau  OR Kenya  OR Lesotho  OR Liberia  OR Libya  OR Madagascar  OR Malawi  OR Mali  OR Mauritania  OR Mauritius  OR Morocco  OR Mozambique  OR Namibia  OR Niger  OR Nigeria  OR Rwanda  OR Sao Tome and Principe  OR Senegal  OR Seychelles  OR Sierra Leone  OR Somalia  OR South Africa  OR South Sudan  OR Sudan  OR Tanzania  OR Togo  OR Tunisia  OR Uganda  OR Zambia  OR Zimbabwe  OR Maghreb*  OR Benin  OR Dahomey  OR Burkina Faso  OR Upper Volta  OR Burundi  OR Urundi  OR Central African Republic  OR Ubangi-Shari  OR Chad  OR Comoros  OR Mayotte  OR "Democratic Republic of Congo"  OR "Democratic Republic of the Congo"  OR "Congo, Dem. Rep"  OR Kinshasa  OR "Belgian Congo"  OR Zaire  OR Katanga  OR Eritrea  OR Ethiopia  OR Gambia  OR "Guinea-Bissau"  OR Guinea  OR Liberia  OR Madagascar  OR "Malagasy Republic"  OR Malawi  OR Nyasaland  OR Mali  OR Mozambique  OR "Portuguese East Africa"  OR Niger  OR Rwanda  OR Ruanda  OR "Sao Tome and Principe"  OR Senegal  OR Sierra Leone  OR Somalia  OR South Sudan  OR Tanzania  OR Zanzibar  OR Tanganyika  OR Togo  OR "Togolese Republic"  OR Uganda  OR Zambia  OR Zimbabwe  OR "Southern Rhodesia"))  NOT TI=("guinea pig*" OR animal)
